# Factors Associated with the Usage of Psychological Support Services for Employees in the Romanian Technology Industry

**DOI:** 10.3390/healthcare13030326

**Published:** 2025-02-05

**Authors:** Mihai Bran, Dragoş Ovidiu Alexandru, Rebeca Sara Chesini, Lavinia Corina Duică, Sanda Amelia Drăcea, Elena Daciana Pintilie, Mihail Cristian Pîrlog

**Affiliations:** 1 Psychiatry Department, Colţea Hospital Bucharest, 030167 Bucharest, Romania; mihaibran@gmail.com; 2 Biostatistics Department, Faculty of Medicine, University of Medicine and Pharmacy of Craiova, 200349 Craiova, Romania; dragos.alexandru@umfcv.ro; 3 Doctoral School, University of Medicine and Pharmacy of Craiova, 200349 Craiova, Romania; rebeca.chesini@umfcv.ro (R.S.C.); daciana.pintilie@umfcv.ro (E.D.P.); 4 Psychiatry Department, Faculty of Medicine, Lucian Blaga University, 550169 Sibiu, Romania; laviniaduica@yahoo.com; 5 Department of Biophysics, University of Medicine and Pharmacy of Craiova, 200349 Craiova, Romania; 6 Medical Sociology Department, Faculty of Medicine, University of Medicine and Pharmacy of Craiova, 200349 Craiova, Romania; mihail.pirlog@umfcv.ro

**Keywords:** mental health services, occupational health, workplace, health services accessibility, motivation, employee health, technology industry, Romania

## Abstract

**Background/Objectives**: Mental health challenges significantly impact employee productivity, especially in high-stress industries like technology. This study aims to investigate the factors influencing the intention to use psychological support services among Romanian tech employees, focusing on barriers and motivators related to availability, accessibility, affordability, and acceptability. **Methods**: A cross-sectional survey was conducted among 372 Romanian tech employees using a structured online questionnaire. The survey assessed socio-demographic data, prior experience with mental health services, perceived distress levels, and barriers categorized into four dimensions. Data were analyzed using descriptive statistics and inferential analysis to identify key predictors of the intention to use these services. **Results**: Key factors influencing the use of psychological support services included prior experience with mental health services, the need to take time off work, and the absence of a companion. Barriers such as cost, transportation, awareness of services, and stigma were not significant. The regression model explained 8.4% of the variance in the likelihood of using these services, suggesting that additional factors may need further exploration. **Conclusions**: Addressing practical barriers, such as time constraints and the absence of support during access, is essential to improving accessibility of these services. Leveraging positive past experiences with mental health services can enhance engagement. These findings can guide the development of targeted interventions to promote the uptake of psychological support services in the tech sector, contributing to a healthier and more productive workforce.

## 1. Introduction

The concept of psychological support in the workplace emerged from the growing recognition of how work-related stress impacts employees’ mental health and overall well-being. Over time, as workplace demands have intensified, with tighter deadlines, longer hours, and increased job insecurity, organizations have come to acknowledge the necessity of supporting employees’ mental health.

Mental health issues in the workforce are a pressing concern, leading to significant economic consequences, including decreased productivity and higher rates of absenteeism [1]. Mental health disorders are among the leading causes of work-related disability, imposing a substantial financial burden on organizations and economies. Globally, depression and anxiety alone account for nearly $1 trillion in lost productivity each year [2]. Beyond productivity losses, these issues drive up healthcare costs, lower job satisfaction, and contribute to higher turnover rates [3]. In particular, the tech industry reports a higher prevalence of mental health disorders among employees compared to the general population, underscoring the urgent need for targeted mental health initiatives in this sector [4].

Investing in workplace mental health programs is not only about enhancing well-being but also offers substantial economic benefits. Studies show that every dollar spent on mental health programs yields an average return on investment (ROI) of 2.3, largely driven by reduced absenteeism and improved productivity [5]. Another analysis reported a median ROI of 4.2 for well-designed and effectively implemented mental health initiatives [6]. The ROI varies depending on factors such as the program’s scope and intensity, the industry, organizational size, and the employees’ baseline mental health status [7].

Employee Assistance Programs (EAPs) were among the earliest workplace psychological support systems, introduced in the United States during the 1940s. Initially focused on providing counselling for employees struggling with substance abuse, EAPs gradually expanded to address mental health and a wide range of personal and work-related challenges. By the 1980s, the concept of occupational stress gained prominence, prompting research into its effects on employee health and productivity. This shift led to the creation of stress management programs aimed at equipping employees with tools to manage stress and improve their mental health.

Workplace mental health programs are essential for fostering a healthy and productive workforce, but their success depends largely on employee participation. Several factors influence the utilization of these programs, including individual attitudes, stigma, organizational culture, and communication strategies [8]. Stigma surrounding mental health can significantly discourage employees from seeking support [9]. Furthermore, some employees may not recognize the need for these services, either due to a lack of awareness or underestimating their own mental health challenges [10].

A supportive organizational culture is key to encouraging program usage. Factors such as management attitudes, workplace stress levels, and norms around help-seeking play a critical role [11]. Accessibility and convenience also affect participation, with flexible options like online or telephone counselling often leading to higher engagement [12]. Clear and effective communication about the program, emphasizing its purpose, benefits, and confidentiality, can further boost utilization [13]. Regular promotion of the program ensures it remains visible and top of mind for employees.

Research suggests that those who would benefit most from counselling are often the least likely to seek it. Thus, it was found that distressed individuals were less inclined to pursue counselling due to anticipated challenges from both internal and external barriers [14]. Exploration of the relationship between emotional competence, defined as the ability to manage emotional difficulties, and the likelihood of seeking psychological help in a survey of 300 undergraduate students, has offered evidence that individuals with lower emotional competence were less likely to seek professional support for managing emotional problems. It was concluded that those most in need of counselling are often the least likely to pursue it [15].

Several factors influence attitudes toward seeking psychological help. Gender differences are notable, with women generally holding more positive views about seeking mental health care compared to men [16,17]. Prior experience with counselling significantly shapes attitudes, fostering greater openness to seeking help [15,18].

Practical barriers such as cost, time constraints, and limited insurance coverage are among the most frequently cited obstacles to accessing mental health care [19,20,21]. Additionally, lack of awareness about available services or uncertainty about where to find them can hinder access. Concerns about being judged or stigmatized, either by oneself or others, represent barriers to the acceptability of services [22]. Accessibility challenges, such as transportation or needing someone to accompany the individual, further complicate help-seeking efforts.

Barriers to seeking counselling can be broadly categorized as emotional or practical [23]. Emotional barriers include discomfort, fear of being overly vulnerable, reluctance to discuss personal issues with strangers, and concerns about others’ opinions. Practical barriers encompass financial costs, time constraints, transportation, and caregiving responsibilities for children or other family members.

Stigma surrounding mental health support significantly affects the utilization of psychological services and it was defined as “the reduction of an individual’s self-esteem or self-worth” resulting from being labelled as “socially unacceptable” [24]. Also, it was highlighted that societal stigmatizing beliefs and attitudes can deter individuals from seeking mental health services or accepting a diagnosis for a psychological or mental health condition. This societal stigma not only influences those considering professional help but also exacerbates challenges for those already receiving care [25].

The impact of mental health conditions over the employees’ performance was presented by previous studies, being reported that up to one in five workers experience mental health challenges annually, ranging from conditions like depression and anxiety to more severe disorders such as bipolar disorder or schizophrenia [26]. In the tech industry, these rates may be even higher due to the unique pressures associated with this field. The tech sector is known for its demanding work environment, which often contributes to mental health concerns among employees. Burnout is a particularly common issue, marked by emotional exhaustion, cynicism, and reduced professional effectiveness. A survey conducted by Blind, a workplace community app, found that 57% of tech employees reported job burnout [27], significantly exceeding the general population burnout rate of 23% [28]. Depression and anxiety are also prevalent among tech workers, and substance use disorders, including alcohol and illicit drug use, are another concern in the tech industry. While comprehensive data are limited, anecdotal evidence suggests that some tech employees use substances as a coping mechanism for high stress levels [29,30,31].

Mental health issues profoundly affect employee productivity and job performance, with existing evidence highlighting that mental health disorders are linked to a 37% increase in absenteeism and a 27% decline in productivity [26]. In the tech sector, these challenges can result in missed deadlines, reduced innovation, and higher turnover rates. Presenteeism, where employees are physically present but unable to function optimally due to mental health struggles, is a significant contributor to decreased productivity [32]. Employees with depression, for example, report notably higher rates of presenteeism, leading to substantial productivity losses [33]. Absenteeism is another costly consequence of poor mental health. Employees with mental health conditions take more days off, and in the U.S., this equates to over 200 million lost workdays annually, costing employers up to $44 billion [1]. In the tech industry, mental health-related absenteeism can severely disrupt project timelines in its fast-paced work environment.

Poor mental health also drives up healthcare costs. Employees with untreated mental health disorders often develop additional physical health conditions, increasing healthcare utilization and expenses [34]. Since tech companies frequently offer health insurance as part of their benefits, these increased costs directly impact their bottom line. Thus, it was estimated that the indirect costs of mental health disorders, including productivity losses and healthcare expenses, are around $17.1 billion annually for U.S. employers. These costs are likely even higher for tech companies due to their competitive nature and high employee salaries [26].

Despite the numerous previous studies performed in this area, there is a notable gap in the existing literature regarding the lack of studies examining the utilization of mental health support services within specific industries, as well as the factors influencing their usage rates. Thus, little attention has been given to strategies for improving employee engagement with these services, even if it was proven that people who could benefit from psychological support may encounter real and imagined obstacles to obtaining treatment. Time, money, and the stigma associated with seeing a mental health specialist are some of the obstacles that are most frequently mentioned in different papers [24,35,36,37]. Additionally, there is limited information regarding specific industries, such as the tech sector.

Romania’s tech industry is expanding rapidly, yet there is little to no data on the availability or utilization of psychological support services for employees in this field. The current study focuses on the usage of psychological support services by employees, measured as their intention to use such services. The analysis explores various independent variables that may impact utilization, including factors related to availability, acceptability, affordability, and accessibility. Other potential factors, such as prior experience with psychological support and the perceived level of distress, are also investigated.

This study aims to explore how these independent variables shape the dependent variable—employee usage of psychological support services—and to offer valuable insights for optimizing these services. By understanding these dynamics, we seek to enhance the integration of mental health support in the Romanian tech industry, improve their return on investment, and provide a comprehensive overview of the factors driving utilization.

## 2. Materials and Methods

The study was developed and implemented in Romania, and it involved employees from technology companies. The technology sector is a developed segment in the Romanian economy with many companies and employees. Recent data suggest that the number of employees in the technology sector is increasing, with more than 16,000 employees year on year, with reports of more than 139,000 employees at the end of 2022 [38]. Based on these numbers, we considered a study sample consisting of male and female respondents who were employees in different technology companies in Romania. The study’s survey responses were gathered using the non-probability sampling approach known as judgement sampling, a kind of purposive sampling, and it is beneficial for exploratory investigations where the sample must adhere to a certain condition [39]. The sample size for this study was based on Cochran’s equation:

$$n_{0}=\frac{Z^{2}pq}{e^{2}}$$

where *e* is the desired level of precision, *p* is the (estimated) proportion of the population which has the attribute in question, and *q* is 1 − *p*. We considered a level of precision of 5%, and a confidence level of 95%, with a 0.5 maximum variability. For these assumptions, the sample size calculated is 385.

The cross-sectional survey was conducted using an online questionnaire in a Google Forms format, distributed to employees of technology companies. The Romanian Association of Software Industry supported the process by contacting its members, who subsequently shared the survey with their employees. No alternative methods of data collection were employed.

The questionnaire was developed and distributed in Romanian. Before its administration, it was pilot-tested with 10 employees from a tech company. Adjustments were made to some questions, primarily to address wording issues, before the survey was launched online.

The online survey was conducted from 10 July to 28 July 2023. By the end of the data collection period, 372 respondents had completed the survey. This response rate was deemed consistent with the proposed sample size and adequate for both statistical analysis and the study’s objectives.

### 2.1. Survey Instrument

The method of the online survey was considered based on reasons such as high sample size, straightforward administration, standardization, data analysis, and objectivity. Previous review of the literature has led to the identification of the key factors that may have an influence the utilization of the psychological services: accessibility, affordability, availability, acceptability, previous experience, and level of distress perceived.

The survey instrument designed for this study included 15 items, the first three assessing socio-demographic factors (age, sex, education level), and the fourth assessed the intention to use psychological support by using a dichotomous “Yes/No” question: “Would you access psychological support services?”.

Participants were asked to rank perceived physical and psychological barriers to receiving psychological support based on factors of availability, accessibility, acceptability, and affordability [22]. Eight barriers to seeking help were listed, with the addition of two new items: ‘Fear of what colleagues might think’ and ‘Other,’ which allowed participants to specify a barrier of their own. Using a Likert scale, participants rated the extent to which their current circumstances prevented them from seeking assistance: 1 for a substantial barrier, 2 for somewhat of a barrier, and 3 for not a barrier.

The constructs of affordability, accessibility, acceptability, and availability, first operationalized by Stefl and Prosperi (1985) [22], have been widely used in help-seeking literature [40,41,42]. These barriers also informed the design of the current study, drawing on prior research [19,37].

The availability factor included 2 items:

- Not knowing services are available;
- Not knowing how to access the services.

The accessibility factor included 2 items:

- No transportation options;
- No one to go with.

The acceptability factor included 3 items:

- Fear of being looked down on;
- Fear of what relatives, friends might think;
- Fear of what colleagues might think.

The affordability factor included 2 items:

- Cost of services;
- Have to take time off from work/school/family.

The barrier ‘Fear of what colleagues might think’ was introduced as an additional factor influencing acceptability. Participants were also given the option to specify a barrier under the category ‘Other.’

Motivators for seeking psychological support were assessed using two items: one evaluated previous experience with psychological support services, and the other assessed the perceived level of distress that would motivate participants to seek help. Previous counselling experience has been identified in several studies [18,42,43] as a practical factor significantly correlated with favorable attitudes toward psychological assistance. To assess this, participants answered a dichotomous ‘Yes/No’ question: ‘Have you ever sought the services of a psychologist or psychotherapist for counselling related to emotional, relational, or psychological difficulties?’

The perceived level of distress was measured using a 10-point Likert scale, which offers a broader range of options and provides more nuanced results. Participants were asked: ‘On a scale from 1 to 10, write down the level of distress that you think would motivate you to seek psychological support’. 

### 2.2. Validity and Reliability

Reliability refers to the consistency of variables used in a study, while validity depends on several factors considered during the research process [44]. Cronbach’s alpha, a commonly used reliability coefficient, measures the internal consistency of items or scales used to assess study variables. A Cronbach’s alpha value greater than 0.7 indicates strong internal consistency. For exploratory studies, a value of 0.6 is also acceptable as a measure of internal consistency [44,45]. Based on the results, all variables used in the survey achieved a Cronbach’s alpha value above 0.7, demonstrating the reliability of the questionnaire items. The overall Cronbach’s alpha value for all items combined was 0.766, further confirming the reliability of the survey.

To ensure validity, the study adapted scales previously validated by earlier researchers, as adaptation is considered a suitable technique for developing research questionnaires [44]. Additionally, a pilot study involving 10 participants was conducted to identify and address any necessary corrections before distributing the questionnaire to the target audience. The pilot study results indicated that the questions were clear, easy to understand, and posed no difficulty for respondents in providing answers.

### 2.3. Data Processing and Analysis

At the end of the data collection period, all responses from Google Forms were ex-ported to Microsoft Excel and checked for errors before analysis. The data were then im-ported into SPSS version 25 (IBM Corp., Armonk, NY, USA) for statistical analysis. Descriptive statistics were used to summarize the demographics of the respondents, while inferential statistics were employed to analyze and present the main findings of the study.

The first stage of the analysis involved the computation of descriptive statistics to provide a comprehensive summary of the demographic characteristics of the respondents. This included the calculation of measures such as frequencies to describe variables like age, gender, and educational background.

To answer the research questions and test the study’s hypotheses, inferential statistics were employed to identify patterns, relationships, and trends within the data. Among the statistical methods used, Kendall’s tau-b (τb) correlation coefficient was selected as a nonparametric measure of association. This test was particularly suited for analyzing the strength and direction of relationships between variables measured on ordinal scales. Kendall’s tau-b is often preferred in such scenarios because it is less sensitive to ties and skewed distributions compared to other correlation coefficients, such as Spearman’s rho.

To ensure the robustness and validity of the statistical models used, a series of assumption tests were conducted prior to performing regression analyses. These tests included assessments for linearity and multicollinearity, both of which are critical for maintaining the quality and interpretability of regression results. Multicollinearity, a condition where independent variables are highly correlated with one another, was also assessed using the tolerance value and Variance Inflation Factor (VIF) were calculated for each independent variable. A tolerance value below 0.1 or a VIF exceeding 10 would indicate significant multicollinearity, which could distort the regression coefficients and reduce the reliability of the model [44].

Following the assumption testing, multivariate logistic regression analyses were performed to address the research questions. This statistical approach was chosen for its ability to model the relationship between a binary dependent variable and multiple independent variables, making it particularly suitable for the study’s objectives.

### 2.4. Ethics Considerations

An ethical approach was taken in conducting the research investigation, adhering to the Helsinki Declaration and other established ethical principles. Respondents were assured of the confidentiality of their survey answers. Only limited personal information, such as gender, age group, and education level, was collected. Additionally, respondents were informed that their responses would be used solely for the purposes of the current study, and no further interpretations or uses of the data would be made. All subjects were included on a voluntary basis. The study was approved by the Ethics Committee of Craiova University of Medicine and Pharmacy of Craiova, Romania.

## 3. Results

The study sample consisted of 372 participants, with a majority being female (266 participants, 71.51%), while males accounted for 106 participants (28.49%), with a mean age of 38.55 + 9.43 years. Regarding age distribution, the largest proportion of participants fell within the 31–40 age group, comprising 148 individuals (39.78%). This was followed by those aged 41–50 years, who made up 109 participants (29.30%). In terms of educational background, over half of the participants (187 individuals, 50.27%) had completed postgraduate studies. Graduate-level education was reported by 154 participants (41.40%), while 31 participants (8.33%) had only a high school education (Table 1).

Regarding their intention to use psychological services, 357 respondents (95.97%) expressed willingness by answering ‘yes’ to the question, ‘Would you access psychological support services?’. We then analyzed the degree of correlation between this expressed intention and other variables included in the questionnaire (Table 2).

The value of the correlation coefficient demonstrated a statistically significant positive relationship between the intention to seek mental health support services and factors such as companion support, fear of colleagues, and available time. Despite the statistically significant results, the correlation coefficients were relatively low, indicating that the relationships between these variables are weak.

Additionally, a multicollinearity test was performed to examine whether there were any strong interrelationships between the independent variables. This test helped assess whether any of the predictors were highly correlated, which could affect the robustness of the regression analysis. The results revealed that while some variables showed low to moderate correlations, there was no indication of severe multicollinearity that would compromise the validity of the model.

### 3.1. The Role of the Independent Variables in Determining the Intention to Use Psychological Services Rates

A multivariate logistic regression analysis was conducted to test the hypotheses and address the research questions. The results of this analysis, including the relationship between the independent variables and the dependent variable, are presented in Table 3. The findings provide insights into how the independent factors contribute to the variation in the dependent variable, offering a comprehensive understanding of the study’s results.

The findings summarized in Table 3 were utilized to address the research questions.

**How does accessibility affect the likelihood of utilizing psychological support services for employees in the Romanian tech companies?**

The accessibility factor was evaluated using the following two items:

- Lack of transportation;
- Lack of a companion.

*Transportation does not affect the intention to use psychological services (H_0_)*. The analysis revealed that transportation had no significant association with the intention to use psychological services (*p* = 0.701). Thus, H_0_ was accepted, indicating that the absence of transportation options does not affect employees’ intentions to access psychological support services.

*Having no one to go with does not affect the intention to use psychological services (H_0_)*. In contrast, the results demonstrated that lacking a companion significantly affects the intention to use psychological services (*p* = 0.048). Consequently, H_0_ was rejected, identifying the absence of a companion as a barrier to accessing psychological support services. This finding suggests that the lack of a companion may impede Romanian tech employees from seeking psychological support.

**How does affordability affect the likelihood of utilizing psychological support services for employees in the Romanian tech companies?**

The affordability factor was assessed using the following two items:

- Cost of services;
- Having to take time off from work, school, or family.

*The cost of services does not affect the intention to use psychological services (H_0_)*. The analysis indicated that the cost of services does not have a significant association with the intention to use psychological services (*p* = 0.732). Therefore, H_0_ was accepted, suggesting that the cost of services does not impact employees’ intentions to access psychological support services.

*Having to take time off from work, school, or family does not affect the intention to use psychological services (H_0_)*. The results showed that the requirement to take time off from work, school, or family significantly affects the intention to use psychological services (*p* = 0.039). Consequently, H_0_ was rejected, identifying this factor as a significant barrier to accessing psychological support services. These findings suggest that for Romanian tech employees, the need to take time away from other responsibilities represents a considerable obstacle to seeking psychological support.

**How does availability affect the likelihood of utilizing psychological support services for employees in the Romanian tech companies?**

The availability factor was evaluated using the following two items:

- Not knowing services are available;
- Not knowing how to access the services.

*Not knowing services are available does not affect the intention to use psychological services (H_0_)*. The analysis revealed that not knowing services are available does not significantly affect the intention to use psychological services (*p* = 0.854). Therefore, H_0_ was accepted, indicating that the lack of awareness about the availability of services is not a barrier to accessing psychological support.

*Not knowing how to access the services does not affect the intention to use psychological services (H_0_)*. The results showed that not knowing how to access the services also does not have a significant association with the intention to use psychological services (*p* = 0.836). Consequently, H_0_ was accepted.

Overall, the findings suggest that neither the lack of awareness about the availability of services nor the lack of knowledge on how to access them constitutes a barrier to the intention to use psychological support services for Romanian tech employees.

**How does acceptability affect the likelihood of utilizing psychological support services for employees in the Romanian tech companies?**

The acceptability factor was assessed using the following three items:

- Fear of being looked down on;
- Fear of what relatives or friends might think;
- Fear of what colleagues might think.

*Fear of being looked down on does not affect the intention to use psychological services (H_0_)*. The analysis indicated that the fear of being looked down on does not significantly affect the intention to use psychological services (*p* = 0.944). Therefore, H_0_ was accepted.

*Fear of what relatives or friends might think does not affect the intention to use psychological services (H_0_)*. The results showed that the fear of what relatives or friends might think does not have a significant association with the intention to use psychological services (*p* = 0.181). Consequently, H_0_ was accepted.

*Fear of what colleagues might think does not affect the intention to use psychological services (H_0_)*. Similarly, the analysis demonstrated that the fear of what colleagues might think does not significantly affect the intention to use psychological services (*p* = 0.095). Thus, H_0_ was accepted.

These findings suggest that stigma-related factors, including the fear of being looked down on and concerns about the opinions of relatives, friends, or colleagues, do not represent significant barriers for Romanian tech employees seeking psychological support services.

**How does previous experience with mental health services affect the likelihood of utilizing psychological support services for employees in the Romanian tech companies?**

*Previous experience with mental health services does not affect the intention to use psychological services (H_0_)*. The analysis demonstrated that previous experience with mental health services was significantly associated with the intention to use psychological services (*p* = 0.001). Therefore, H_0_ was rejected, indicating that prior engagement with mental health services is a relevant factor influencing employees’ decisions to seek psychological support. These findings highlight that having previous experiences with mental health services serves as a significant motivator for employees to utilize psychological support services.

**How does the level of distress perceived affect the likelihood of utilizing psychological support services for employees in the Romanian tech companies?**

*The perceived level of distress does not affect the intention to use psychological services (H_0_)*. The analysis indicated that the perceived level of distress does not significantly affect the intention to use psychological services (*p* = 0.901). Therefore, H_0_ was accepted, suggesting that the level of distress experienced by employees does not affect their intention to access psychological support services.

### 3.2. Answering the Major Research Question

The major research question of the study was: **‘What are the key factors influencing the usage of psychological support services for employees in Romanian tech companies?’**

To answer this question, a multiple regression analysis was conducted. The results include the r value, r^2^, and adjusted r^2^, which indicate the goodness of fit for the regression model in explaining the relationship between independent variables and the rate intention to use psychological services. The model and the measured outcome, the intention to use psychological services, revealed a low positive relationship (r = 0.290). The r^2^ value indicates that only 8.4% of the intention to use psychological support services can be explained by the factors included in the model: previous experience, service availability, accessibility, transportation, companion support, feelings of being looked down upon, fear of family, fear of colleagues, cost, time, and distress level.

The stepwise multiple regression procedure was conducted to group the independent variables with the most significant impact on the intention to use psychological support services. At each step, the independent variable with the smallest probability of F is added, provided that the probability is sufficiently low. Variables currently in the regression equation are removed if their probability of F becomes sufficiently high. This process continues until no further variables are eligible for inclusion or removal.

For the new model, the predictors are:

- Have to take time off from work/school/family (time);
- Have you ever sought the services of a psychologist or psychotherapist for counselling related to emotional, relational or psychological difficulties (previous experience);
- No one to go with (companion).

The second model and the measured outcome show a low positive relationship (r = 0.268). The r^2^ value indicates that only 7.2% of the intention to use psychological support services can be explained by the factors included in the model: time, previous experience, and companion.

Based on the results of our analysis, we identified several key factors that significantly shape the intention to address psychological services among Romanian tech employees. The first and most prominent factor is the challenge of balancing personal and professional responsibilities, specifically the difficulty of taking time off from work, school, or family obligations to attend psychological sessions. Another factor is previous experience with psychological services. Employees who have engaged with a psychologist or psychotherapist in the past tend to be more open to seeking such support again. This suggests that familiarity and positive prior interactions can shape their willingness to access these services. Lastly, the feeling of isolation or the lack of someone to accompany them when accessing psychological services emerged as a significant deterrent. The absence of a supportive companion can make individuals feel hesitant or anxious about attending sessions, further contributing to underutilization.

### 3.3. Analysis of the Role of Gender and Age in the Intention to Use Psychological Support Services

As a basis for the further development of our study, we conducted a multivariate analysis to examine gender and age differences in the intention to use psychological support services. We assumed that *gender does not affect the intention to use psychological support services*. *(H_0_)*. According to the data presented in Table 4, H_0_ was accepted. Gender does not significantly affect the intention to use psychological services (*p* = 0.156). The hypothesis that *age does not affect the intention to use psychological support services (H_0_)* was rejected, indicating that age is a factor that shape the intention to use psychological support services.

## 4. Discussion

In this study, we explored the role of accessibility in the intention to use psychological services among Romanian tech employees. Accessibility was measured through two factors: no transportation and no one to go with. Among these, only the variable ‘no one to go with’ significantly affects employees’ willingness to utilize psychological support services. Transportation, on the other hand, does not significantly alter employees’ decision to seek psychological support. Previous studies have shown that both transportation and companionship are critical determinants of individuals’ decisions to access psychological services [22]. The results of this study could be attributed to the growing availability of online solutions, such as platforms that offer live video counselling sessions, especially after the COVID-19 pandemic. These platforms provide a convenient and accessible way for individuals to seek psychological support without the need for physical transportation or the presence of a companion, which can facilitate easier access to mental health services. IT professionals in particular rely heavily on the internet as their primary source of information for various aspects of daily life, including their physical and mental health needs [46] and this amount of high-quality mental health information and online resources can have a profound impact on their health outcomes [47].

Regarding the affordability, represented by two items (cost of services and having to take time off from work/school/family), we demonstrated that for our study population have partially affected the decision to seek psychological support. If some other studies [22,48,49,50,51] highlighted the fact that cost was the main deterrent to getting professional assistance, our results have proven that for the Romanian tech employees, the cost of the services was not a factor affecting their decision to seek psychological support. This finding could be explained by the fact that psychological support services are offered as a benefit by the companies, so they are free of charge, respectively, that the incomes in the tech sector are above the Romanian average income so they can afford to pay for the psychological services.

When looking at the time component, meaning that employees have to take time from work/school/family, this factor appeared to significantly affect their decision to use psychological support services. The finding is similar to the results of previous studies [22,51,52,53]. Since time is a factor that shapes the intention to use the support services, a solution to optimize the time of employees looking for support services could be to change the design of mental support programs and interventions.

Our analysis of the availability factor, which included two specific elements, lack of awareness about available services and lack of knowledge about how to access these services, revealed no significant association with the rates of intention to use psychological services among Romanian tech employees. Neither of the sub-factors, “not knowing that services are available” nor “not knowing how to access the services”, demonstrated any measurable consequence on employees’ decisions to seek psychological support.

This suggests that awareness and access, while traditionally considered barriers, are not primary concerns for this particular population in the context of utilizing psychological services. Interestingly, this result contrasts with findings from previous research which identified these availability-related factors as significant barriers to accessing mental health services [22,52,54]. The divergence in findings may point to changes in service accessibility, improved communication strategies within organizations, or differences in cultural or occupational contexts over time.

The study investigating the role of the acceptability factor on decisions to seek psychological support services found no significant outcome. According to our methodology, the acceptability factor was measured through three items (fear of being looked down on; fear of others’ opinions—relatives and friends; fear of colleagues’ perceptions), which represent emotional barriers, often described as stigma [23]. Emotional barriers involve intrapersonal judgments shaped by concerns about being perceived as weak, flawed, or vulnerable by others. Prior studies suggest that these factors are significant barriers to accessing care [9,41,55]. A potential explanation for the findings in our study is that the participants were well-educated. Research indicates a negative correlation between educational attainment and the stigma associated with psychological problems. Educated individuals may perceive less stigma, reducing the impact of emotional barriers on their decision to seek support [56,57].

The results of the analysis demonstrated that prior experience with mental health services significantly affects the likelihood of utilizing psychological support services. Individuals who have previously engaged in counseling are more inclined to seek psychological assistance, as this prior experience serves as a practical motivator, helping in reducing uncertainty about the process, making it easier to recognize the benefits of professional help. This positive relationship between past counseling experience and favorable attitudes toward seeking psychological assistance is well-documented in the literature. Other studies provided evidence that individuals with previous exposure to counseling often display less hesitation and more openness when considering mental health services. Their findings suggest that prior counseling helps to normalize the experience and mitigate concerns about stigma or unfamiliarity [18,42,43]. Moreover, prior counseling experience may foster trust in mental health professionals and reduce perceived barriers such as fear of judgment or doubts about effectiveness. By gaining firsthand knowledge of the process, individuals often feel more confident about seeking help in the future. This highlights the importance of initiatives that encourage initial engagement with mental health services, as even a single experience can positively shape future attitudes and behaviors toward seeking psychological support [58].

One of the aims of this study was to investigate the role of perceived distress levels among Romanian tech employees on their intention to utilize psychological support services. The analysis revealed that perceived distress levels did not significantly alter the intention to use such services. This finding contrasts with evidence from other research, which argue that high levels of psychological distress may compel individuals to prioritize their mental health needs over concerns about breaking culturally prescribed norms [59,60]. In such cases, distress acts as a critical driver, pushing individuals to seek help despite potential stigma or discrimination [61,62,63,64,65]. However, in the current study, neither the perceived distress levels nor stigma associated with seeking psychological support appeared to significantly shape Romanian tech employees’ decisions to access these services. A possible explanation is that they may have developed alternative coping mechanisms for managing stress, such as reliance on peer networks, workplace resources, or personal resilience strategies, or that the relatively low stigma levels reported among this group, combined with moderate distress levels, may have created a neutralizing effect.

The impact of the two socio-demographic elements considered in analysis, gender and age, on the intention to seek psychological support services was different. The findings revealed that gender does not significantly affect the likelihood to use psychological services. This result contrasts with prior research, which consistently suggests that women typically expressing greater willingness to engage in psychological support [55,66,67]. If in earlier studies, socialization patterns were considered as motivators for women to express vulnerability and seek help. In the context of the current study, the absence of a gender factor might reflect shifts in attitudes toward help-seeking within the studied population, particularly among Romanian tech employees. Our results could also point to increasing gender equality in attitudes toward mental health or the specific workplace factors, such as the tech industry’s emphasis on problem-solving and innovation, may contribute to normalizing help-seeking behaviors across genders. In contrast, age was found to significantly affect the intention to seek psychological support services. This aligns with previous research, which has observed variations in help-seeking behaviors across age groups being shown that younger individuals are often more open to using psychological services compared to older adults. Younger people may have greater exposure to mental health awareness campaigns and less stigma attached to seeking psychological support, while older adults may rely more on informal support networks or view seeking help as a last resort [18,40,68,69,70,71]. These findings suggest that interventions aimed at promoting mental health services should consider the distinct needs of different age groups.

This study examined factors influencing the willingness to use psychological support services among Romanian tech employees, integrating both barriers and motivators into a conceptual framework. The model developed can be applied in future research to explore help-seeking behaviors across different industries, employee groups, or countries. The results contribute to understanding the factors that shape employees’ use of psychological services, enabling comparisons across industries and populations. Also, companies integrating psychological support services can use these insights to optimize human resource strategies, focusing on time-efficient programs and leveraging employees with prior counselling experience to promote the benefits of such services. Mental health services providers can refine their offerings by addressing barriers, tailoring services by age group, and improving accessibility. Incorporating the study’s findings will help better meet client needs and drive business growth.

Overall, this research provides a foundation for improving the design and delivery of psychological support services, benefiting researchers, employers, and service providers alike.

### Limitations of the Study

This study provides valuable insights for researchers and practitioners, though several limitations should be noted.

First, one potential limitation is the reliance on self-reported data, which can introduce measurement errors due to biases such as social desirability or recall bias. Moreover, the cross-sectional design of the study limits the ability to make causal inferences, as it only captures data at one point in time, preventing the examination of temporal relationships between variables.

Second, the sample size fell slightly short of the target required by Cochran’s formula. While 385 respondents were needed, only 372 surveys were completed, which may impact the statistical power of the findings. The use of convenience sampling also introduces certain limitations, primarily related to generalizability. As participants were selected based on accessibility rather than randomization, the sample may not fully represent the broader population, potentially leading to selection bias.

Third, only 8.4% of the intention to use psychological support services could be explained by the factors used in the model: previous experience, service available, how to access, transportation, companion, looked down, fear of family, fear of colleagues, cost, time, and distress level. This suggests that other, unexamined factors may play a significant role for employees in the Romanian tech sector and should be investigated further.

Fourth, the survey respondents were predominantly from large Romanian tech companies already familiar with psychological support services. Romania’s tech industry also includes numerous small- and medium-sized enterprises with fewer resources and employees. Future studies should aim to include participants from a broader range of company sizes to ensure more representative findings.

Additionally, the study focused exclusively on Romanian tech industry employees, which limits the generalizability of the conclusions to other industries or regions. Expanding future research to compare employees across various sectors and European countries would provide more comprehensive insights applicable to diverse business environments and cultural contexts.

## 5. Conclusions

This study identified key factors influencing the intention to use psychological support services by Romanian tech employees. The presence of a companion when accessing services; the need to take time off from work, school, or family; and prior experience with mental health services significantly impacted employees’ decisions to seek psychological support. Specifically, the absence of a companion and the requirement to take time off were identified as substantial barriers, while prior experience with mental health services emerged as a strong motivator. In contrast, factors such as transportation, service costs, lack of awareness about service availability or access, fear of being looked down on, fear of judgment from relatives or colleagues, and perceived distress levels did not affect the willingness of using psychological services among this population. Thus, these factors are not perceived as barriers or motivators by Romanian tech employees.

These findings highlight the importance of addressing specific barriers, such as the need for support during access and time constraints, while leveraging prior counseling experiences to encourage greater utilization of psychological services among Romanian tech employees.

## Figures and Tables

**Table 1:** Socio-demographic data.

Variable	Categories	*n* (%)
Gender	Male	106 (28.49)
	Female	266 (71.51)
Age	18–25 years	23 (6.18)
	26–30 years	51 (13.71)
	31–40 years	148 (39.78)
	41–50 years	109 (29.30)
	51–60 years	31 (8.33)
	61–70 years	10 (2.69)
Education	High school	31 (8.33)
	Graduate studies	154 (41.40)
	Postgraduate studies	187 (50.27)

**Table 2:** The correlations between the intention to use psychological services and other variables included in the questionnaire.

	Kendall Tau-b	*p*
Have you ever sought the services of a psychologist or psychotherapist for counselling related to emotional, relational or psychological difficulties?	0.160	**0.0009**
Not knowing services are available	−0.048	0.294
Not knowing how to access the services	−0.035	0.445
No transportation	0.028	0.554
No one to go with	0.173	**0.0002**
Fear of being looked down on	0.029	0.541
Fear of what relatives, friends might think	0.039	0.407
Fear of what colleagues might think	0.139	**0.004**
Cost of services	0.065	0.156
Have to take time off from work/school/family	0.154	**0.0008**
On a scale from 1 to 10, write down the level of distress felt that you think would motivate you to seek psychological support	−0.071	0.095

**Table 3:** Coefficients of independent variables on the intention to use psychological support service rates.

Model	Unstandardized Coefficients		Standardized Coefficients	t	Sig.
Dependent Variable: Intention to Seek Mental Health Support Services	B	Std. Error	Beta		
(Constant)	1.705	0.074		22.912	0.000
Have you ever sought the services of a psychologist or psychotherapist for counselling related to emotional, relational or psychological difficulties?	0.071	0.021	0.183	3.336	**0.001**
Not knowing services are available	−0.003	0.017	−0.013	−0.185	0.854
Not knowing how to access the services	−0.004	0.017	−0.016	−0.207	0.836
No transportation	0.007	0.017	0.026	0.385	0.701
No one to go with	0.050	0.025	0.137	1.985	**0.048**
Fear of being looked down on	0.002	0.026	0.006	0.070	0.944
Fear of what relatives, friends might think	−0.043	0.032	−0.135	−1.341	0.181
Fear of what colleagues might think	0.049	0.029	0.163	1.674	0.095
Cost of services	−0.005	0.015	−0.021	−0.343	0.732
Have to take time off from work/school/family	0.031	0.015	0.126	2.074	**0.039**
On a scale from 1 to 10, write down the level of distress felt that you think would motivate you to seek psychological support	−0.001	0.005	−0.007	−0.125	0.901

**Table 4:** The role of gender and age in the intention to use psychological services.

**Model**		**Sum of Squares**	**df**	**Mean Square**	**F**	**Sig.**
Gender	Regression	0.074	1	0.074	2.024	0.156
	Residual	12.432	340	0.037		
	Total	12.506	341			
Age	Regression	0.222	1	0.222	6.172	**0.013**
	Residual	12.287	342	0.036		
	Total	12.509	343			

## Data Availability

The data used in this study will be available upon reasonable request and subject to approval from the local IRB.
